# Molecular insights into the multifaceted functions and therapeutic targeting of high mobility group box 1 in metabolic diseases

**DOI:** 10.1111/jcmm.17448

**Published:** 2022-06-15

**Authors:** Zhipeng Tao, My N. Helms, Benjamin C. B. Leach, Xu Wu

**Affiliations:** ^1^ Cutaneous Biology Research Center, Harvard Medical School Massachusetts General Hospital Boston Massachusetts USA; ^2^ Pulmonary Division, Department of Internal Medicine University of Utah Salt Lake City Utah USA

**Keywords:** advanced glycation end products, autophagy, genomic stability, inflammation, toll‐like receptors

## Abstract

HMGB1 is a ubiquitously expressed protein localized in nucleus, cytoplasm, as well as secreted into extracellular space. Nuclear HMGB1 binds to DNAs and RNAs, regulating genomic stability and transcription. Cytoplasmic HMGB1 regulates autophagy through binding to core autophagy regulators. Secreted extracellular HMGB1 functions as a ligand to various receptors (RAGE and TLRs, etc.), regulating multiple signalling pathways, such as MAPK, PI3K and NF‐κB signallings. Trafficking and localization of HMGB1 across cellular compartments could be regulated by its posttranslational modifications, which fine‐tune its functions in metabolic diseases, inflammation and cancers. The current review examines the up‐to‐date findings pertaining to the biological functions of HMGB1, with focus on its posttranslational modifications and roles in downstream signalling pathways involved in metabolic diseases. This review also discusses the feasibility of targeting HMGB1 as a potential pharmacological intervention for metabolic diseases.

## 1 INTRODUCTION

The high mobility group (HMG)‐box (HMGB) family belongs to the HMG protein superfamily that plays important roles in modulating chromatin structures. The HMGB family consists of four chromosomal proteins: HMGB1, HMGB2, HMGB3 and HMGB4. HMGB1 is ubiquitously expressed in high abundance (roughly 1 × 10^6^ molecules per mammalian cell), whereas HMGB2, 3 and 4 are expressed at lower levels with restricted expression patterns.^1^, ^2^, ^3^ Herein, we summarize the literature reports on the roles of HMGB1 in metabolic diseases and discuss the perspectives of targeting HMGB1 as a potential therapeutic approach.

HMGB1 protein is both a nuclear factor and a secreted protein.^4^ In the nucleus, HMGB1 serves as a transcriptional regulator that binds or bends DNAs or RNAs and promotes transcriptional complex assembly on specific gene targets.^5^, ^6^ For example, HMGB1 binds to the promoter region of *TNF* and promotes the assembly of the repressor NF‐κB factor RelB, thus suppressing TNFα expression.^7^ Upon infection or injury, HMGB1 has been shown to translocate from nucleus to the cytoplasm and could also be secreted by activated immune cells.^8^ In addition, HMGB1 can be secreted by various cells, including hepatocytes, keratinocytes and granulocytes, under necrosis, DNA damage or oxidative stress.^9^, ^10^, ^11^, ^12^ Once secreted, HMGB1 functions as a ligand and binds to the receptor for advanced glycation end products (RAGE), toll‐like receptors (TLRs) and C‐X‐C Motif Chemokine Receptor 4 (CXCR4), playing important roles in regulation of inflammation and immune responses.^13^

**FIGURE 1:**
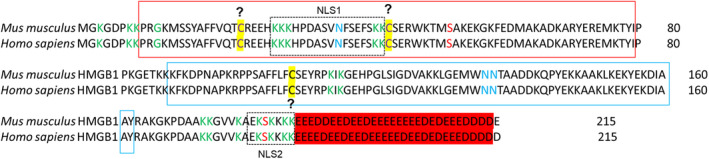
Primary sequence of HMGB1 and its posttranslational modification sites. Human HMGB1 consists of 215 amino acids, which comprises A‐box (red solid box), B‐box (blue solid box) and acidic tail (red background). HMGB1 is phosphorylation at Ser39, Ser53 and Ser181. HMGB1 is N‐glycosylated at Asn37, Asn134 and Asn135. Redox sensitivity and disulfide linkages that may occur at amino acid positions of Cys23, Cys45 and Cys106 in HMGB‐1 require additional evaluation, as indicated by ‘?’. HMGB1 is hyperacetylated at multiple sites of lysine in two nuclear localization sequences (NLS). Green colour indicates acetylation sites, red colour indicates phosphorylation sites, blue colour indicates glycosylated sites, and purple colour indicates disulfide bound sites. Yellow background indicates oxidized and reduced sites. Question marks indicate site modifications needed re‐evaluation. Dashed boxes indicate the NLS domains

HMGB1 consists of 215 amino acids, organized into two high mobility group boxes, which function as DNA‐binding domains and an acidic C‐terminus, respectively (Figure 1). It has been reported that several serine residues of HMGB1, including Ser39, Ser53 and Ser181, could be phosphorylated by protein kinase C zeta (PKC ζ), enhancing HMGB1 secretion.^14^ HMGB1 is also hyperacetylated at multiple lysine residues near the nuclear localization sequence (NLS) (Figure 1), and such posttranslational modification of lysine residues is affected by JAK/STAT1 signalling.^11^, ^15^, ^16^ The molecular mechanism responsible for dynamic acetylation and deacetylation of HMGB1 remains unclear and requires further investigation. Poly(ADP‐ribose) polymerase‐1 (PARP‐1)‐induced poly(ADP‐ribosyl)ation of HMGB1 synergizes with acetylation and ultimately leads to HMGB1 secretion under LPS stimulation.^11^ In addition, HMGB1 could be *N*‐glycosylated at Asn37, Asn134 and Asn135, which is critical for promoting HMGB1 secretion.^17^ However, inter‐plays between different posttranslational modifications of HMGB1 have not been fully studied, which warrant further investigation.

## 2 REDOX REGULATION OF HMGB1

There are three conserved cysteine residues (Cys23, Cys45 and Cys106) on HMGB1 that have been implicated as redox‐sensitive regulators of HMGB1’s pro‐inflammatory activity. In general, all protein Cys residues with functional thiol groups [containing a sulphur and hydrogen atom (‐SH)] could be posttranslationally modified upon oxidative stress. In silico structural analysis of HMGB1 predicts that Cys23 and Cys45 could reversibly form intramolecular disulfide bonds^18^ that can be cleaved by glutaredoxin‐catalysed, GSH‐dependent reduction. Under severe oxidative stress, Cys‐SH groups in proteins can be hyperoxidated to sulfinic [SO_2_H] and then sulfonic acids [SO_3_H], and such modifications have been shown to promote protein degradation. Whether hyperoxidation of HMGB1 confers its pro‐inflammatory function remains unclear. Previous reports have suggested that oxidation‐specific epitopes on HMGB1 could be differentially recognized by TLR and RAGE receptors. However, such claims have been found unreliable, and the reports were retracted recently.^19^, ^20^, ^21^, ^22^, ^23^ Although independent proteomic analyses of HMGB1 support the notion that disulfide bond formation between Cys residues confers HMGB1’s pro‐inflammatory functions,^24^ the overall strength of this body of literatures linking oxidized HMGB1 to pro‐inflammatory responses remains low and unconvincing. There remains a critical need to rigorously assess the redox state(s) of HMGB1 in order to (re)‐evaluate the signal transduction and biological activities stimulated by HMGB1 that occur under reducing and oxidizing conditions.

In summary, subcellular localization and secretion of HMGB1 direct its biological functions, which is tightly regulated by its posttranslational modifications, including phosphorylation,^14^ acetylation,^12^ *N*‐linked glycosylation^17^ and Poly(ADP‐Ribosyl)ation.^11^, ^16^ The regulation of HMGB1 in inflammation and immunity has been systemically reviewed.^13^ Therefore, the current review will focus on the role of HMGB1 in metabolic diseases, given the fact that inflammation also plays a critical in the initiation, propagation and development of metabolic diseases.^25^

## 3 NUCLEAR FUNCTION OF HMGB1 IN THE REGULATION OF METABOLIC DISEASE

**FIGURE 2:**
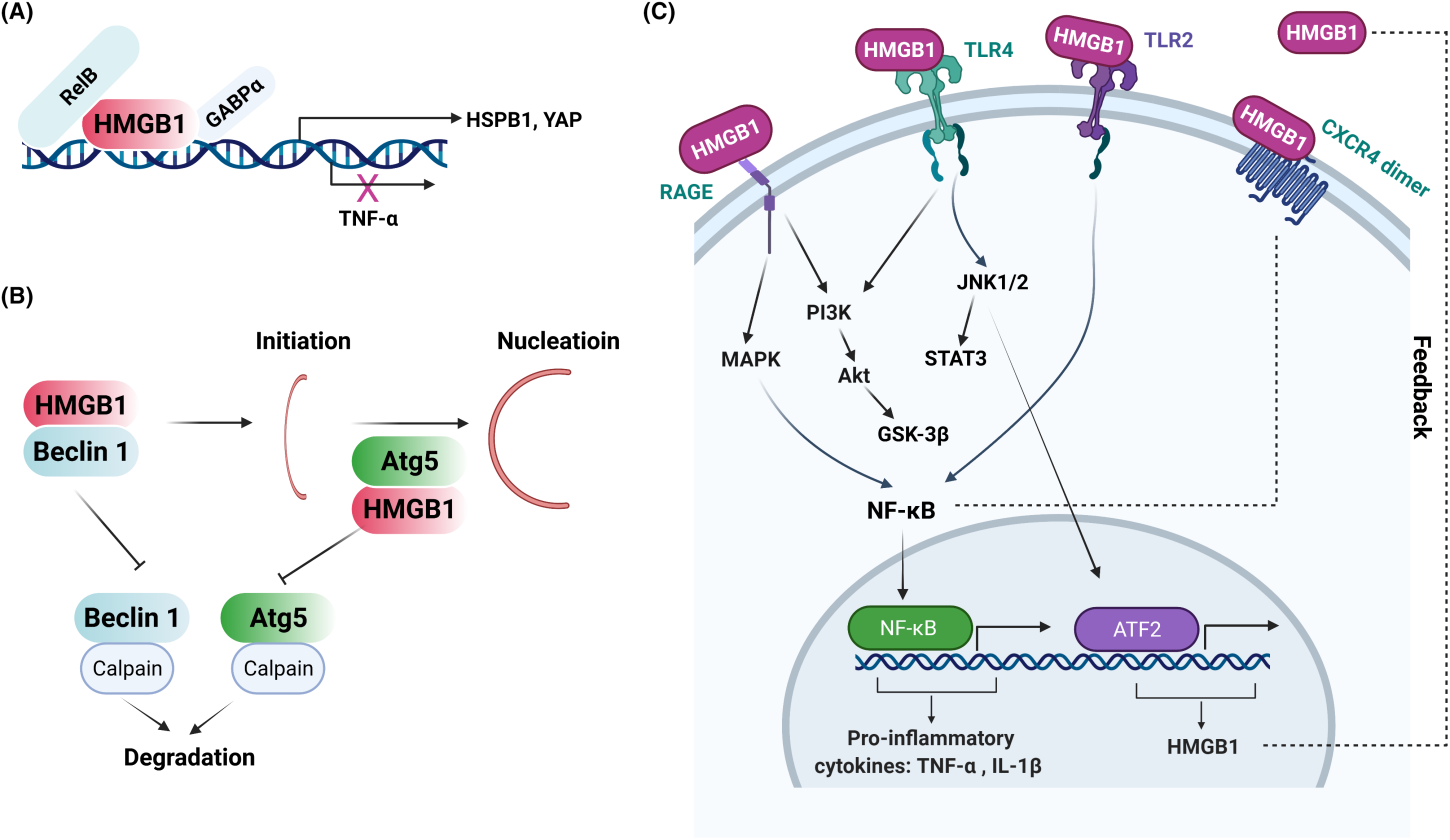
Schematic diagram of HMGB1’s function in the cytoplasm, nucleus and extracellular spaces. (A) In the nucleus, HMGB1 binds (such as promotors of *YAP*, *HSBP1*) and bends DNA, which maintains genome stability and regulates gene transcriptions. (B) In the cytoplasm, HMGB1 binds to protein partners and facilitate various cellular function, such as the binding of Beclin1 in the process of autophagy. (C) In the extracellular space, HMGB1 binds to several receptors, such as TLR2, TLR4 and RAGE, and regulates downstream signalling pathways, including MAPK, PI3K, NF‐κB and JAK/STAT, which are essential in signalling network of diseases

In the nucleus, HMGB1 can bind to or bend with the DNAs or RNAs with its transcriptional partners, and controls gene transcription and affect downstream targets, such as *YAP* (yes‐associated protein (1), *HSPB1* (Heat shock protein beta‐1) and *TNFA*. Many of these target genes have previously been associated with metabolic diseases^7^, ^26^, ^27^ (Figure 2A).

For example, HMGB1 promotes diethylnitrosamine (DEN)‐induced liver dysfunctions and tumorigenesis through its impact on YAP promoter and increased expression of YAP target genes, such as *BIRC5*, *CCND1, MYC, SPP1* and *GPC3*.^26^ Mechanistically, HMGB1 binds to GABPα, an important transcription regulator of *YAP* gene expression, and HMGB1‐GABPα complex binds to the *YAP* promoter (Figure 2A). Knockout of HMGB1 reduces cell proliferation but does not cause cell death or DNA damage. Consistently, HMGB1 knockout reduces the expression of core components of the Hippo pathway (e.g., *YAP* and *WWTR1*/*TAZ*) and YAP/TAZ target genes, such as *BIRC5, CCND1, MYC, SPP1* and *GPC3*, independent of Wnt/‐catenin and Notch pathways, suggesting that nuclear HMGB1 could affect cell proliferation through regulation of YAP/TAZ expression.

Warburg effect, also known as aerobic glycolysis, is a metabolic hallmark of most cancer cells, including HCC, characterized by an excessive conversion of glucose to lactate, despite sufficient oxygen supply.^28^ HMGB1‐YAP axis has been shown to be responsible for the Warburg effect in liver tumorigenesis via stabilization of YAP‐HIF1α in the nucleus and enhancing HIF1α DNA‐binding activity, leading to aerobic glycolysis and Warburg effect^26^ (Figure 2A). In addition, HMGB1 regulates the expression of heat shock protein beta‐1 (HSPB1), which is essential for maintaining quality control of mitochondria.^27^ Mechanistically, HMGB1 increases HSPB1 expression, which controls actin cytoskeleton and delivers damaged mitochondria for degradation by autophagy (i.e., mitophagy).^27^ Recent studies have demonstrated HMGB1 as a bona fide RNA‐binding protein affecting splicing choices.^6^ Together, HMGB1 functions as a DNA‐ or RNA‐binding protein, modulating gene expression, splicing and translation, which are relevant in many pathological conditions. Further studies are warranted to specify which DNA or RNA sequences are bound with HMGB1 and their physiological and disease relevance.

## 4 CYTOPLASMIC FUNCTIONS OF HMGB1 IN METABOLIC DISEASES

Cytoplasmic HMGB1 has been shown to associate with proteins involved in mitochondrial metabolism, such as mitochondrial degradation (mitophagy) and quality control, and autophagy. Dysregulation of these processes has been implicated in metabolic diseases.^29^ For example, HMGB1 is responsible for the suppressive effects of p53 on autophagy in nonalcoholic fatty liver disease (NAFLD). It has been shown that translocation of HMGB1 from nucleus to cytoplasm, and subsequent induction of Beclin1 expression play important roles in NAFLD.^30^ Mechanistically, HMGB1 interacts with Beclin1 and Atg5, which are key components for autophagic initiation and nucleation^31^ (Figure 2B). The complex of HMGB1‐Beclin1 and HMGB1‐Atg5 are resistant to degradation by calpain, inhibiting mitochondria‐dependent cell death.^32^, ^33^ As autophagy is a process underlying many human diseases,^34^ it would be interesting to further study the roles of HMGB1 on autophagy in metabolic diseases.

## 5 EXTRACELLULAR FUNCTION OF HMGB1 IN METABOLIC DISEASES

Previously, the functions of extracellular HMGB1 in metabolic diseases have been widely studied. Secreted HMGB1 could bind to receptors, such as RAGE, toll‐like receptor 2 and 4 (TLR2/4), and CXCR, regulating downstream signalling, including MAPK, PI3K and NF‐κB **(**Figure 2C).^13^, ^35^, ^36^ All these regulatory events are highly relevant to metabolic diseases, such as diabetes and associated complications, liver diseases (e.g., nonalcoholic steatohepatitis [NASH] and liver injury) and cardiovascular diseases.

HMGB1 is implicated in the development of diabetes and hyperglycemia, and various associated complications in brain, lung, kidney and bones. For example, serum HMGB1 is a predictive biomarker of type 2 diabetes mellitus (T2DM) and chronic obstructive pulmonary disease (COPD). Studies have shown that serum HMGB1 levels are positively correlated with HOMA‐IR, fasting plasma glucose (FBG) and glycated haemoglobin (HbA1c), and negatively correlated with lung functions in subjects with both T2DM and COPD.^37^ Interestingly, clinical data indicate that the serum HMGB1 levels are not associated with gestational diabetes mellitus (GDM), although HMGB1 was correlated with maternal age, a risk factor of GDM.^38^ In addition, HMGB1 plays a critical role in diabetes‐related dysfunctions of bone marrow stromal cells (BMSCs) and impaired osteointegration.^39^ The regulatory role of secreted HMGB1 in diabetes and associated complications, as well as liver disease and cardiovascular diseases, has been shown to be through its binding to different receptors.

Obstructive sleep apnoea (OSA), a common T2DM complication, leads to exacerbated intermittent hypoxia (IH), which could severely affect cognitive functions.^40^ IH induces HMGB1‐TLR4 signalling in hippocampal tissue, concomitant with suppressed autophagy and enhanced apoptosis.^41^ Silencing of HMGB1 suppressed TLR4 signalling, and restored autophagy and suppressed apoptosis in hippocampal neurons of animal model of T2DM with OSA complication.^41^ Activation of microglial cells may account for neuronal apoptosis and cognitive deficits.^42^ For example, in one recent study, diabetic KK‐Ay mice exhibited increased cognitive dysfunction, microglial activation and hippocampal neuronal apoptosis, compared with C57 control mice.^42^ Activation of BV2 microglia leads to active secretion of HMGB1 from microglial cells and the secreted HMGB1 functions as an inflammatory factor and sustains the activation of these microglial cells in a positive feedback loop, leading to deteriorated neuronal damage. Mechanistically, HMGB1 activates NF‐κB‐p65 in microglia, resulting in the secretion of TNF‐α and IL‐1β, and excessive ROS, which mediates the apoptosis of HT22 cells via the PI3K/Akt/GSK‐3β signalling pathway **(**Figure 2C).^42^ Moreover, extracellular HMGB1 is involved in streptozotocin‐induced diabetic nephropathy (DN) via its activation of TLR2, TLR4 and RAGE, while blockade of HMGB1 signalling attenuates streptozotocin‐induced DN.^43^ Furthermore, HMGB1 plays a crucial role in diabetic neuropathy accompanied neuroinflammation, characterized by the upregulation of HMGB1 and its receptors (TLR4 and CXCR4) **(**Figure 2C).^35^ In addition, periodontal inflammation in diabetic patients was regulated by HMGB1‐RAGE‐TNF‐α/IL6 signalling.^44^ However, the regulation of HMGB1 in inflammatory response and cytokine secretion is context (cell or tissue) dependent. For example, hyperglycaemia‐triggered trophoblast cell secretion of IL‐8 and anti‐migratory response is dependent on HMGB1‐TLR4 signalling, while IL‐1β production was HMGB1‐TLR4 signalling independent.^45^

In a high‐fat diet (HFD)‐induced NAFLD model, HMGB1 expression and extracellular release are potentiated by a saturated fatty acid (palmitic acid) in vitro, whereas the neutralization of endogenous HMGB1 dampens HFD‐induced inflammatory responses and impairment of liver functions in vivo.^46^ HMGB1 expression is controlled by JNK1/2–ATF2 axis transcriptionally, and the miR‐200 family of microRNAs post‐transcriptionally. The upregulation and release of HMGB1 could, in turn, self‐activate TLR4–JNK1/JNK2–ATF2 signalling, thus forming a positive feedback loop **(**Figure 2C).^46^ Furthermore, clinical studies have revealed that HMGB1 is relevant in colonic inflammation and inflammatory bowel disease (IBD)‐like phenotypes with NAFLD.^47^ Mechanistically, HMGB1 is secreted from liver in NAFLD, which induces an inflammatory response, through binding to its receptors (RAGE and TLR4), and regulation of redox signalling, leading to ectopic intestinal inflammation.^48^ Similarly, previous studies indicated that intestinal epithelial cells (IEC) secrete HMGB1, further elevating enteric inflammation in NAFLD following local injury.^49^ Ablation of HMGB1 leads to lipid accumulation in jejunal IEC, decreases chylomicron packaging and/or release, reduces serum triglycerides (TG) and mitigates liver steatosis, thus preventing HMGB1 ^ΔIEC^ mice from high‐fat, high‐cholesterol and fructose‐enriched diet (HFCFD) induced‐NASH.^49^ In the liver tissue of streptozotocin‐induced diabetic rats, HMGB1‐TLR4 signalling is activated, accompanied by the activation of MAPK (p38, ERK, JNK), NFκB‐p65 and JAK1/STAT3 signalling pathways, which contributes to systemic inflammation **(**Figure 2C).^50^ Hyperhomocysteinemia (HHcy) induces expression and secretion of HMGB1 in vascular endothelial cells, which is regulated by Neuropilin‐1 (NRP1)‐p38 MAPK axis.^51^ Vascular endothelial cell inflammation and dysfunction are common complications of diabetes that is controlled by hyperglycaemia‐induced miR‐106 and subsequent induction of HMGB1 expression and inflammation.^52^

Taken together, extracellular HMGB1 is involved in the development of metabolic diseases, mainly through its cytokine‐like effects. Thus, targeting extracellular HMGB1 might provide potential therapeutic agents for metabolic diseases.

## 6 PHARMACOLOGICAL INTERVENTIONS OF HMGB1 IN METABOLIC DISEASE

As HMGB1 plays important roles in metabolic diseases, significant efforts have been directed towards the discovery of specific HMGB1 inhibitors.^53^ A recent report has expatiated the findings of pre‐clinical HMGB1‐directed therapies. Such therapeutic approaches involve direct binding of small molecule/peptide‐like antagonists to HMGB1 and ultimately result in the inhibition of posttranslational modifications and/or neutralization of extracellular HMGB1.^53^ In addition, colonic epithelial hyperplasia is detected in the small intestines of DM mice, which is an underlying risk factor for colorectal cancer development.^54^ HMGB1 and RAGE levels are elevated in *db/db* type 2 diabetic mouse model and high glucose‐treated NCM460 colon cells, while butyrate suppresses the abnormal proliferation of colonic epithelial cells under diabetic state by targeting HMGB1‐RAGE.^54^ Hyperglycaemia (HG) exacerbates myocardial ischaemia/reperfusion (I/R) injury with the activation of HMGB1‐RAGE/TLR2/TLR4‐NF‐κB signalling, which is reversed by ethyl pyruvate (EP) treatment.^55^ In addition, EP treatment in mice significantly suppresses streptozotocin‐induced type 1 diabetes (T1D) development, in line with the reduced infiltration of cells into the pancreatic islets, enhanced regulatory T cell (Treg) and suppressed IFN‐γ‐producing cells, leading to preservation of β‐cell functions.^56^ Mechanistically, EP has been shown to mitigate the pro‐inflammatory activity of HMGB1 in NASH, suppressing the activation of MAPK (p38, ERK, JNK), NF‐κB‐p65 and JAK1/STAT3 signalling pathways and protecting the diabetic liver from injury. In addition, another HMGB1‐specific inhibitor, glycyrrhizin reverses the upregulation of HMGB1 and its receptors (TLR4 and CXCR4) in mice with diabetic neuropathy, improving mechanical and thermal pain threshold in these animals. Mechanistically, glycyrrhizin inhibits the release of HMGB1.^35^ Moreover, in vitro and in vivo studies of glycyrrhizin demonstrate that it improves osteogenic differentiation, attenuates lipid peroxide, restores hyperglycaemia‐induced impairment of trabecular structure and osteointegration, via inhibiting HMGB1‐RAGE cascade.^39^ Together, the therapeutic interventions on HMGB1 are mainly related to their targeting of extracellular HMGB1. However, it would be also critical to design specific drugs targeting extracellular or nuclear HMGB1 for different utilities.

## 7 CONCLUSIONS AND PERSPECTIVE

HMGB1 plays multifaced roles in metabolic diseases through its differential functions at different subcellular locations. In the nucleus, HMGB1 functions as transcriptional regulator, bending and binding with DNAs or RNAs. In the cytoplasm, HMGB1 is an autophagy or mitophagy regulator through its binding to autophagic components. In the extracellular space, HMGB1 plays distinct roles via its binding to various receptors and regulates downstream signalling pathways, which are critical mediators of metabolic diseases. Recently, validity of studies about redox regulation of HMGB1 has been questioned; therefore, extra cautions should be exercised to consider the redox modifications of HMGB1 and its functions.

Since posttranslational regulations of HMGB1 have not been fully studied, more efforts are needed to explore the posttranslational modifications of HMGB1 under normal physiological and metabolic diseases conditions. Such efforts might lead to the development of specific drugs targeting HMGB1‐associated metabolic diseases. Given the critical roles of HMGB1 in maintaining genomic stability and binding (and bending) to DNAs and RNAs, further studies are warranted to elucidate more HMGB1 nuclear partners and their direct target genes in physiological and pathological conditions.

## AUTHOR CONTRIBUTIONS

**Zhipeng Tao:** Conceptualization (equal); investigation (equal); project administration (equal); supervision (equal); writing – original draft (equal); writing – review and editing (equal). **Xu Wu:** Conceptualization (equal); investigation (equal); project administration (equal); resources (equal); supervision (equal); writing – original draft (equal); writing – review and editing (equal). **Benjamin C.B. Leach:** Writing – review and editing (supporting). **My N. Helms:** Conceptualization (equal); investigation (equal); supervision (equal); writing – original draft (equal); writing – review and editing (equal).

## CONFLICT OF INTEREST

The authors declare that they have no conflict of interest.

## Data Availability

Data sharing not applicable to this article as no datasets were generated or analysed during the current study.
